# Histone Deacetylases and Histone Deacetylase Inhibitors in Neuroblastoma

**DOI:** 10.3389/fcell.2020.578770

**Published:** 2020-10-07

**Authors:** Monica Phimmachanh, Jeremy Z. R. Han, Yolande E. I. O’Donnell, Sharissa L. Latham, David R. Croucher

**Affiliations:** ^1^The Kinghorn Cancer Centre, Garvan Institute of Medical Research, Sydney, NSW, Australia; ^2^St Vincent’s Hospital Clinical School, University of New South Wales, Sydney, NSW, Australia

**Keywords:** neuroblastoma, histone deacetylases, histone deacetylase inhibitor, acetylation, differentiation, apoptosis, cell cycle arrest

## Abstract

Histone deacetylases (HDACs) are enzymes that play a key role in regulating gene expression by remodeling chromatin structure. An imbalance of histone acetylation caused by deregulated HDAC expression and activity is known to promote tumor progression in a number of tumor types, including neuroblastoma, the most common solid tumor in children. Consequently, the inhibition of HDACs has emerged as a potential strategy to reverse these aberrant epigenetic changes, and several classes of HDAC inhibitors (HDACi) have been shown to inhibit tumor proliferation, or induce differentiation, apoptosis and cell cycle arrest in neuroblastoma. Further, the combined use of HDACi with other chemotherapy agents, or radiotherapy, has shown promising pre-clinical results and various HDACi have progressed to different stages in clinical trials. Despite this, the effects of HDACi are multifaceted and more work needs to be done to unravel their specific mechanisms of actions. In this review, we discuss the functional role of HDACs in neuroblastoma and the potential of HDACi to be optimized for development and use in the clinic for treatment of patients with neuroblastoma.

## Introduction

Neuroblastoma is a highly malignant pediatric tumor that arises within the sympathetic nervous system. It is the most common extracranial solid tumor in children, accounting for 7–10% of all pediatric cancers and 15% of pediatric cancer-related deaths (Maris et al., 2007). While neuroblastoma patients are classified into low, intermediate and high-risk groups, according to disease stage, patient age and specific genetic mutations (Papaioannou and McHugh, 2005), the majority of patients present with advanced stage, high-risk disease. Despite the use of high-intensity treatment regimens incorporating multi-agent chemotherapy, radiotherapy and immunotherapy, these high-risk patients continue to have poor clinical outcomes, with therapy-resistant relapse occurring in up to 60% of cases (Maris et al., 2007; Ora and Eggert, 2011; Tonini et al., 2012).

Although the overall mutational burden in neuroblastoma is very low compared to other cancer types (Schramm et al., 2015), high-risk disease is often characterized by amplification of the oncogenic driver *MYCN* (Schwab et al., 1983). The encoded protein, N-Myc, promotes neuroblastoma tumorigenesis by driving the expression of genes involved in cell proliferation, and suppressing those required for differentiation and apoptosis (Domingo-Fernandez et al., 2013; Fey et al., 2015). While there are currently no therapeutic options available to directly target N-Myc activity, alternative strategies have emerged to indirectly regulate N-Myc-mediated transcription, including epigenetic modulation via HDAC inhibition (Fletcher et al., 2018; Jubierre et al., 2018). Along with the emergence of HDACs as drivers of drug resistance in neuroblastoma (Keshelava et al., 2007; Oehme et al., 2009, 2013; Lodrini et al., 2013), there has been a considerable effort to investigate the use of HDACi as treatment strategies for high-risk neuroblastoma (Jubierre et al., 2018). Therefore, this review focuses on the role of HDACs and HDACi in neuroblastoma and advances the understanding of how HDACi can disrupt multiple cancer pathways, resulting in single-agent activity, as well as synergistic combinations with other anti-cancer agents.

### Histone Modifications

**FIGURE 1:**
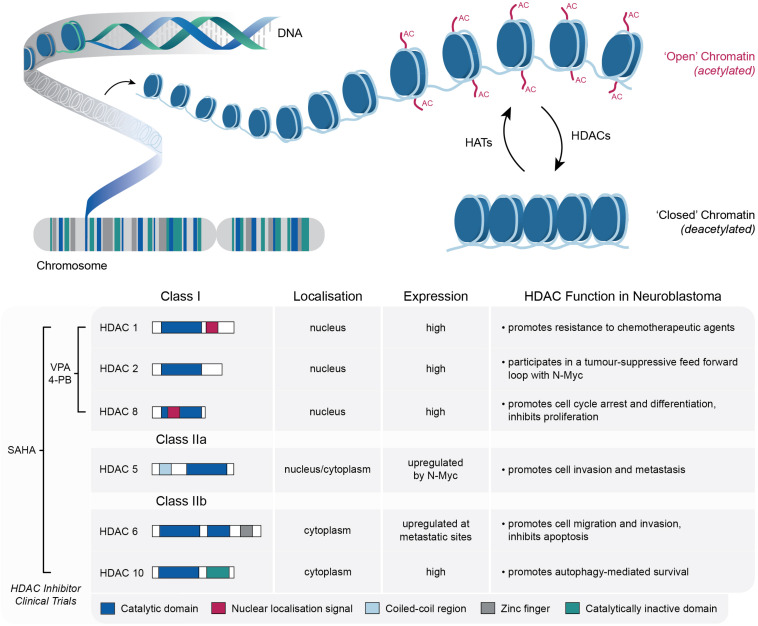
Schematic representation of the role of HATs and HDACs in the dynamic modification of lysine acetylation within histone tails, which mediates the switching between “open” (relaxed) and “closed” (condensed) chromatin structures. Details of the specific HDACs implicated in neuroblastoma tumorigenesis are also shown, along with the relevant HDAC inhibitors that have been utilized in neuroblastoma clinical trials.

As central DNA scaffolding proteins, the post-translational modification of histones plays a key role in regulating chromatin conformation, which ultimately modulates the accessibility of DNA to the transcriptional machinery (Bannister and Kouzarides, 2011; Waldmann and Schneider, 2013; Audia and Campbell, 2016; Lawrence et al., 2016). These post-translational modifications include acetylation, methylation, phosphorylation and sumoylation; each of which is regulated by enzymes that facilitate either the addition or removal of these chromatin marks (Bolden et al., 2006; Zhao and Shilatifard, 2019). A key example of this is the opposing activity of histone acetyltransferases (HATs) and HDACs, which is known to tightly regulate gene expression by altering chromatin structure between relatively “open” and “closed” states (Tang et al., 2013). HATs transfer acetyl groups to a number of lysine residues in histones H2A, H2B, H3, and H4, resulting in the local expansion of chromatin and increased accessibility of regulatory proteins to DNA, whereas HDACs catalyze the removal of acetyl groups, which in turn drives chromatin condensation and transcriptional repression (Thiagalingam et al., 2003; Wapenaar and Dekker, 2016; Figure 1). Both enzymes are important in normal cellular physiology, although an imbalance in the equilibrium of histone acetylation has been associated with tumorigenesis and cancer progression in a number of tumor types, including neuroblastoma (Gronbaek et al., 2007; Iacobuzio-Donahue, 2009; Pfister and Ashworth, 2017).

## HDACs

Deemed master regulators of gene expression, HDACs are involved in regulating a number of biological processes including apoptosis, cell cycle progression and differentiation (Xu et al., 2007). Aside from primarily targeting histone proteins, more than 50 non-histone targets of HDACs have also been discovered (Glozak et al., 2005). The human HDAC family consists of 18 enzymes that are subdivided into four classes based on their homology to yeast HDACs, subcellular localization and enzymatic activities (Bolden et al., 2006). Class I HDACs (1, 2, 3, and 8) contain a deacetylase domain and show homology to the yeast protein RPD3. They are expressed in the nuclei of most cell types and are involved in the transcriptional repression of a number of genes. Class II HDAC members are subdivided into two classes—class IIa HDACs (4, 5, 6, 7, and 9) and class IIb HDACs (6 and 10). These HDACs are homologous to yeast Hda1 and unlike class I HDACs, are not limited to the nucleus. Class IIa HDACs are distinguished by the presence of an N-terminal extension, whilst class IIb HDACs comprise two deacetylase domains. In the case of HDAC6, this second deacetylase domain is reportedly responsible for the deacetylation of non-histone targets, including the cytoskeletal protein a-tubulin (Yang and Gregoire, 2005). Class III HDACs, also known as Sirtuins (SIRT 1-7), rely on NAD^+^ cofactors and are homologs of the yeast protein Sir2. HDAC11, the latest and lone member of class IV is the smallest isoform of the HDAC family, sharing features of both Class I and II HDACs (Bolden et al., 2006; Clocchiatti et al., 2011).

### The Role HDACs in Neuroblastoma

In several cancers, the aberrant expression of HDACs largely correlates with tumor onset, progression and global histone hypo-acetylation (Fraga et al., 2005; Bolden et al., 2006). In fact, a clear association between HDAC activity, tumor growth and cell survival has been well established in a broad spectrum of hematologic and solid tumors (Richon et al., 1998; Marks and Xu, 2009). As outlined below, in the setting of neuroblastoma a number of Class I and Class II HDACs have been implicated in promoting tumor progression, cell motility or drug resistance (Figure 1).

#### Class I HDACs

A comparison of gene expression profiles between two drug sensitive and three multidrug-resistant neuroblastoma cell lines by Keshelava et al. (2007) previously identified HDAC1 as a candidate gene for conferring multidrug resistance. RNA expression analysis across a large panel of neuroblastoma cell lines further demonstrated that significantly higher HDAC1 mRNA levels were present in multi-drug resistant lines compared to drug-sensitive lines. Functionally, selective knockdown of HDAC1 sensitized the multi-drug resistant CHLA-136 cell line to etoposide, a topoisomerase II inhibitor commonly used for the treatment of high-risk neuroblastoma (Keshelava et al., 2007).

Investigations of the expression levels of classical HDACs across a large cohort of primary neuroblastoma samples also identified HDAC8 as a prognostic indicator of advanced disease stage and poor survival (Oehme et al., 2009). Here, upregulated expression of HDAC8 in advanced, metastatic disease was associated with poor prognostic markers such as 1p and 11q deletions, age (>18 months) and an unfavorable Shimada histopathology score. Interestingly, HDAC8 was downregulated in stage 4S neuroblastoma cases, which are known to undergo spontaneous regression (Oehme et al., 2009). These clinical findings were further supported by *in vitro* assays demonstrating that HDAC8 knockdown inhibited the proliferation of BE(2)-C, SK-N-BE, Kelly, and SH-SY5Y cells, as well as inhibiting clonogenic growth and promoting cell cycle arrest and differentiation in BE(2)-C cells (Oehme et al., 2009).

Amplification of the proto-oncogene MYCN is well established as an adverse prognostic marker in neuroblastoma (Domingo-Fernandez et al., 2013; Lodrini et al., 2013). Functionally, its oncogenic potential is in part facilitated through the recruitment of HDAC2 in a forward loop to transcriptionally repress the tumor suppressive miR-183 in neuroblastoma cells (Lodrini et al., 2013). Lodrini et al. (2013) conducted an RNAi-mediated screen of 11 class I, IIa, IIb, and IV HDACs and found that specific HDAC2 depletion caused an increased in miR-183 expression, whilst HDAC2 overexpression conversely reduced miR-183 levels. Furthermore, HDAC2 depletion also enhanced histone H4 pan-acetylation, indicating increased transcriptional activation. These results suggest a novel way to target MYCN-amplified tumors may be through HDAC2 inhibition (Lodrini et al., 2013), which is further supported by other studies revealing that HDAC2 knockdown promoted apoptosis in BE(2)-C cells (Oehme et al., 2009).

#### Class II HDACs

HDAC5, a member of the class IIa HDAC family, has previously been implicated in promoting the invasion and metastasis of neuroblastoma (Fabian et al., 2016). This effect was thought to be mediated through the transcriptional repression of the tetraspanin CD9, mediated by the binding of both HDAC5 and N-Myc to the CD9 promoter. In line with evidence from ovarian (Furuya et al., 2005) and bladder carcinoma cell lines (Mitsuzuka et al., 2005), elevated CD9 expression was found to suppress neuroblastoma cell migration and invasion, whilst low CD9 tetraspanin expression within primary neuroblastomas correlated with *MYCN* amplification, high-risk disease and poor patient survival. Although CD9 expression could be elevated by either HDAC5 siRNA, or treatment with the HDACi panobinostat, across a number of neuroblastoma cell lines, siRNA mediated knockdown of HDACs 2, 3, and 10 elicited opposing effects on CD9 expression. Whilst these data demonstrate the potential for indirect therapeutic targeting of CD9 via the inhibition of HDAC5, the development of a more specific HDAC5 inhibitor may be of benefit in this context.

Members of the class IIb HDAC sub-family, HDAC6 and HDAC10 contain two deacetylase domains and can shuttle between the nucleus and the cytoplasm (Yang and Gregoire, 2005). In addition to histones, HDACs have been shown to deacetylate various non-histone substrates. For instance, α-tubulin, heat shock protein 90 (HSP90), and cortactin are several non-histone substrates of HDAC6 that are critical in regulating cell proliferation, metastasis, invasion, and mitosis in tumors (Li et al., 2018). In neuroblastoma, inhibition of HDAC6 can enhance cell adhesion and impair both polarization and efficient migration, which correlates with findings that HDAC6 expression is upregulated at metastatic sites, such as the mediastinum, abdominal cavity, and pelvic cavity (Zhang et al., 2014). Additionally, HDAC6 is also thought to contribute to neuroblastoma tumorigenesis through regulating Bax-dependent apoptosis via deacetylation of Ku70 and regulation of the interaction between Ku70 and Bax (Subramanian et al., 2011). These results suggest that HDAC6 upregulation results in enhanced cell motility and invasiveness, while also improving tumor survival by suppressing apoptosis through Ku70 deacetylation.

Further studies also suggest that the class IIb HDAC sub-family member, HDAC10 may exert specific roles in neuroblastoma progression, as elevated HDAC10 expression has previously been associated with poor clinical outcomes in high risk neuroblastoma patients (Oehme et al., 2013). Functionally, HDAC10 promoted autophagy-mediated cell survival through deacetylation of autophagy related 4D cysteine peptidase (ATG4D), which in turn regulates autophagosome formation and increases autophagy flux (Oehme et al., 2013). In line with this, elevated HDAC10 promoted resistance to doxorubicin, while its depletion restored sensitivity of drug resistant cells to doxorubicin treatment (Oehme et al., 2013).

## HDAC Inhibitors in Neuroblastoma

Given the critical role of HDACs in various cancers, including neuroblastoma, there has been a considerable effort to pursue the use of small molecule HDACi in a therapeutic setting. A number of different HDACi have been developed over the last couple of decades, which can be classified into six groups based on their chemical structure (Table 1). This includes a number of class-specific HDAC inhibitors, although some are considered “pan-HDAC” inhibitors, which are structurally diverse and display inhibitory activity across all isoforms of the zinc-dependent HDAC classes with little discrimination. A number of pre-clinical studies have assessed the anti-tumor effects of these HDACi in neuroblastoma, in general highlighting their ability to inhibit cell proliferation, while promoting cell cycle arrest, differentiation and apoptosis (Table 1). Consequently, the safety and efficacy of a small number of HDACi have also been evaluated within neuroblastoma clinical trials (Table 2).

**TABLE 1:** HDAC inhibitors used in Neuroblastoma focused pre-clinical studies.

**Drug name**	**HDAC Class specificity**	**Observed functional effects**	**References**
**Short-chain fatty acids**			
Valproic Acid (VPA)	Class I and IIa	Growth Inhibition *in vitro* and *in vivo* Differentiation Apoptosis Synergistic in combination with celecoxib, ABT-510 and OGX-011	Cinatl et al., 2002; Rocchi et al., 2005; Stockhausen et al., 2005; Yang et al., 2007; Hrebackova et al., 2009; Liu et al., 2009; Chen et al., 2011
Sodium Butyrate	Class I and IIa	Growth Inhibition *in vitro* Differentiation Apoptosis	Rocchi et al., 1992; De los Santos et al., 2007; Muhlethaler-Mottet et al., 2008; Lorenz et al., 2011
Tributyrin	Class I and IIa	Growth Inhibition *in vitro* Differentiation	Rocchi et al., 1998
Sodium Phenylbutyrate (4-PB)	Class I and IIa	Growth Inhibition *in vitro* and *in vivo* Apoptosis Inhibition of DNA synthesis Synergistic in combination with vincristine	Pelidis et al., 1998; Tang et al., 2004
**Hydroxamic acids**			
m-carboxycinnamic acid bis-hydroxamide (CBHA)	Class I	Growth Inhibition *in vivo* Apoptosis Effective in combination with retinoids	Coffey et al., 2001
Vorinostat (SAHA)	Class I, II, and IV	Growth Inhibition *in vitro* Differentiation Apoptosis Cell cycle arrest Sensitize resistant cells Downregulation of stemness genes Effective in combination with retinoids	De los Santos et al., 2007; Muhlethaler-Mottet et al., 2008; Lautz et al., 2012; Zheng et al., 2013
Panobinostat (LBH-589)	Class I, II, and IV	Growth Inhibition *in vitro* and *in vivo* Differentiation Apoptosis Synergistic in combination with cisplatin, doxorubicin or etoposide	Wang et al., 2013; Waldeck et al., 2016
Abexinostat (PCI-24781)	Class I and II	Growth Inhibition *in vitro* and *in vivo* Apoptosis Effective in combination with bortezomib	Sholler et al., 2013
Trichostatin A (TSA)	Class I, II, and IV	Growth Inhibition *in vitro* Apoptosis Cell cycle arrest Autophagy	De los Santos et al., 2007; Hrebackova et al., 2009; Francisco et al., 2012
BL1521	Class I and II	Growth Inhibition *in vitro* Differentiation Apoptosis	de Ruijter et al., 2004, 2005; Ouwehand et al., 2005
Tubacin	Class IIb (HDAC6)	Impairs polarized morphology and impedes migration	Zhang et al., 2014
1-naphthohydroxamic acid (Cpd2)	Class I (HDAC 8)	Growth Inhibition *in vitro* and *in vivo* Differentiation Apoptosis Effective in combination with retinoic acid	Rettig et al., 2015
PCI-34051	Class I (HDAC 8)	Growth Inhibition *in vitro* and *in vivo* Differentiation Apoptosis Effective in combination with retinoic acid	Rettig et al., 2015
**Benzamides**			
Entinostat (MS-275)	Class I	Growth Inhibition *in vitro* and *in vivo* Apoptosis Inhibition of DNA synthesis Cell cycle arrest Morphological Changes Effective in combination with acetazolamide	Jaboin et al., 2002; Bayat Mokhtari et al., 2017
M344	Class I and II	Growth Inhibition *in vitro*	Furchert et al., 2007
**Cyclic peptides**			
Romidepsin (Depsipetide/FK228)	Class I	Growth Inhibition *in vitro* and *in vivo* Apoptosis Sensitizes multi-drug resistant cells to cytotoxic agents	Keshelava et al., 2007; Panicker et al., 2010
Helminthosporium carbonum (HC)-toxin	Class I	Growth Inhibition *in vitro* Differentiation Apoptosis Cell cycle arrest Represses colony formation Inhibits invasive growth	Deubzer et al., 2008a,b
**Trifluoromethyl ketone**			
HKI 46F08	Class I and II	Growth Inhibition *in vitro* Apoptosis Differentiation Represses colony formation	Wegener et al., 2008
**Ortho-amino anilides**			
BRD840	Class I	Differentiation Effective in combination with retinoic acid	Frumm et al., 2013)
**Sirtuin inhibitors**			
Cambinol	Class III	Growth Inhibition *in vitro* and *in vivo* Anti-tumor activity Cell cycle arrest	Marshall et al., 2011; Lautz et al., 2012

**TABLE 2:** Overview of selected HDAC inhibitors in clinical investigations.

**Drug name and dose**	**Combination agent**	**Rationale for combination**	**Phase and clinical trial identifier**	**Study dates**
Valproic Acid (VPA)–5 mg/kg	Temsirolimus (mTOR Inhibitor)	Minimal, non-overlapping toxicities. VPA has also shown *in vitro* and *in vivo* anti-tumor effects in wide range of pediatric cancers. *In vitro* additive effects of both agents against neuroblastoma (Coulter et al., 2013)	Phase I–NCT01204450	Nov 2009–Mar 2013
Vorinostat (SAHA)–180 mg/m^2^ (maximum dose 400 mg)	Isotretinoin (Retinoid)	Isotretinoin is a standard of care retinoid employed to treat high-risk neuroblastoma. Several pre-clinical studies highlighting the benefits of combination therapy (Table 1; Pinto et al., 2018).	Phase I–NCT00217412 NCT01208454	Aug 2005–Sep 2009 Dec 2010–Sep 2014
	Bortezomib (Proteasome Inhibitor)	Targeting the proteasome-dependent pathways with bortezomib and the aggresome pathway with HDACi results in the accumulation of poly-ubiquitinated proteins, which increases cell stress and apoptosis (Muscal et al., 2013).	Phase I–NCT01132911	May 2010–Apr 2011
	Radiation: 131I-MIBG	Sensitizing effects of SAHA to ionizing radiation have been demonstrated in a metastatic neuroblastoma xenograft model. SAHA also increases uptake of the norepinephrine transporter (NET) allowing for increased MIBG accumulation. Non-overlapping toxicity profiles have also been noted (DuBois et al., 2015).	Phase I–NCT01019850 Phase II–NCT02035137	Mar 2010–Feb 2015 Jul 2014–Jul 2020
	Immunotherapy +/− DFMO	Significant and sustained (5 years) survival benefits were seen in an immunotherapy combination regimen with GM-CSF, IL-2 and isotretinoin in a multinational, phase III study. Despite this, serious adverse reactions have been reported with the immunotherapy-containing regimen and so combinations with HDACi may provide a lower toxicity profile, lowering the chance of risks or other complications (Hoy, 2016; Ozkaynak et al., 2018).	Phase I–NCT02559778	Sep 2015–Sep 2026 (Estimated)
Sodium Phenylbutyrate (4-PB)–410 mg/kg		Selective for HDAC class I and II, several pre-clinical trials have noted the effectiveness of 4PB in neuroblastoma, reducing proliferation, inducing differentiation and impairing tumor growth and metastasis *in vitro* and *in vivo*. Cytotoxic in combination with vincristine *in vitro* (Pelidis et al., 1998) and enhances the expression of favorable marker genes (Tang et al., 2004).	Phase I–NCT00001565	Dec 1996–Oct 2000

One of the most extensively assessed HDACi in neuroblastoma is Valproic acid (VPA). With a higher affinity for Class I HDACs, VPA has been shown to strongly inhibit tumor cell proliferation, apoptosis and induce morphological differentiation, as indicated by an increase in neurite extensions and upregulation of neuronal markers (Rocchi et al., 2005; Stockhausen et al., 2005). Despite showing promise as a single-agent treatment, several studies have also highlighted the therapeutic benefits of combining VPA with other structurally diverse compounds. For instance, VPA was found to exert synergistic cytotoxic effects in combination with celecoxib, an FDA-approved COX-2 inhibitor that has shown chemotherapeutic potential in various cancer settings (Chen et al., 2011). Further, combinations of VPA with the angiogenic inhibitor, ABT-510, and the clusterin inhibitor, OGX-011, have also been shown to impair tumor growth in neuroblastoma xenograft models (Yang et al., 2007; Liu et al., 2009). In 2009, a phase 1 trial was initiated to evaluate the efficacy of combination therapy with VPA and the mTOR inhibitor temsirolimus, in young patients with multiple relapsed solid tumors, including neuroblastoma. However, this trial was terminated due to a lack of funding without reaching the original estimated enrolment numbers.

Another well studied HDACi in neuroblastoma is vorinostat (Suberanilohydroxamic Acid -SAHA), a broad spectrum HDACi that targets both class I and II HDACs and has been successful in phase II trials with FDA approval for use in patients with cutaneous T-cell lymphoma (Duvic et al., 2007; Bubna, 2015). In the neuroblastoma SH-SY5Y cell line, vorinostat treatment increased histone H3 acetylation at Lys^9^ and Lys^14^, and induced extensive apoptotic cell death (De los Santos et al., 2007). Moreover, vorinostat strongly impaired the hypoxia-induced secretion of VEGF, potentiating its anti-angiogenic effect (Muhlethaler-Mottet et al., 2008). In drug resistant MYCN-amplified cells, vorinostat treatment also increased sensitivity to chemotherapy, reduced *in vitro* invasion and downregulation of genes associated with stem-cell behavior (Zheng et al., 2013). Furthermore, vorinostat has been shown to sensitize neuroblastoma cells to various therapeutic modalities, including the pan-Cdk inhibitor flavopiridol (Huang et al., 2010), retinoic acid (De los Santos et al., 2007; Hahn et al., 2008) and radiation (Mueller et al., 2011). Together, these findings demonstrate the clinical potential of vorinostat as a combination therapy for the treatment of neuroblastoma, and underline the rationale for many of the clinical trials evaluating vorinostat in this disease context (Table 2).

4-phenylbutyrate (4-PB), another class I and II HDACi, has also been tested in clinical trials for the treatment of recurrent malignant gliomas (Phuphanich et al., 2005) and neuroblastoma (Table 2). Experimentally, 4-PB suppressed the growth of subcutaneous neuroblastoma xenografts in mice, which was accompanied by an increase in apoptosis and the elevated expression of favorable prognostic markers including EPHB6, EFNB2, EFNB3, NTRK1, and CD44 (Tang et al., 2004). Combination studies have also shown that 4-PB has an additive cytotoxic effect with vincristine *in vitro* (Pelidis et al., 1998).

### Proposed Mechanisms of Action

The attractiveness of combination therapy with HDACi has resulted in a number of clinical trials implemented to evaluate the safety and efficacy of HDACi with standard of care, targeted therapies, radiation and immunotherapies (Table 2). The rationale for these approaches usually involved reported synergistic effects in pre-clinical studies, along with minimal to low overlapping toxicity being observed between combined agents, allowing for increased patient tolerability. Unfortunately, the lack of reporting from these clinical trials prevents a detailed evaluation of the benefit of these drug combinations. Although an important aspect to consider for this combinational approach would be the future design of rational, data driven combination trials that take into account a detailed mechanism of action of each agent. As outlined above, HDACs can promote a number of different, and potentially opposing cellular functions in neuroblastoma cells. Therefore, a consideration of both the desired outcome of HDAC inhibition, as well as an understanding of the specificity required to achieve this outcome, will be required for the development of effective combination therapies, which is a standard therapeutic approach required for neuroblastoma patients (Fletcher et al., 2018).

#### Differentiation

Neuroblastoma is known to arise from sympathetic neuronal precursor cells that were unable to complete the process of differentiation, and several clinical observations have noted a remarkable spontaneous differentiation and regression of these tumors (Hedborg et al., 2010; Brodeur, 2018). A wealth of pre-clinical studies have demonstrated the ability of retinoids, including all-trans-retinoic acid (ATRA, tretinoin) and 13-cis-retinoic acid (isotretinoin), to promote the differentiation of neuroblastoma cells, leading to the adoption of retinoid therapy as a widely employed treatment strategy for neuroblastoma patients (Reynolds et al., 2003; Ora and Eggert, 2011).

Retinoic acids bind to Retinoic Acid Receptors (RARs), which are ligand-regulated nuclear hormone receptors. RARs heterodimerise with Retinoid X Receptors (RXRs) and exert wide ranging effects on transcription by binding to retinoic acid response elements within promoter regions (Siddikuzzaman et al., 2011). In the absence of ligand, RAR/RXR heterodimers repress transcription through the recruitment of corepressors and HDACs (Chen and Evans, 1995). Following retinoic acid binding, a conformation change results in an exchange of this multi-protein complex for either a HAT-containing coactivator complex (Chen et al., 1997), or a ligand-dependent corepressor complex containing alternative HDACs (Fernandes et al., 2003).

The administration of retinoic acid to neuroblastoma cells can often result in distinct morphological changes typical of differentiated neurons, including an increase in out-branched, neurofilament positive neurites (Reynolds et al., 2003). This altered morphology is the result of broad transcriptional changes that promote the elevated expression of a suite of differentiation related genes (Duffy et al., 2017), and is also associated with activation of the PI3K, mitogen-activated protein kinase (MAPK) and Wnt signaling pathways (Lopez-Carballo et al., 2002; Qiao et al., 2012; Duffy et al., 2017). Despite the clinical benefit of retinoid therapy, treatment has proved ineffective for many high-risk patients due to MYCN-induced resistance (Reynolds et al., 2003). Therefore, it has been proposed that this form of differentiation therapy may play a complementary role in combination therapies for neuroblastoma, rather than as single agent therapy.

Given the involvement of HDACs in mediating the transcriptional effects of RARs, it is perhaps unsurprising that several HDACi have been shown to induce extensive morphologic and metabolic changes in neuroblastoma cells that are indicative of differentiation (Table 1). While it is unclear whether HDACi can promote a fully committed, irreversible differentiation of neuroblastoma cells, their use as candidates for differentiation therapy either as single agents or in combination with retinoids is of significant interest. Studies that have provided a mechanistic insight into the process of HDACi induced differentiation include those with Cpd2 and PCI-34051, selective inhibitors of HDAC8. Promisingly, when used in combination with retinoic acid, these inhibitors induced elongated, neurofilament-positive neurites in both BE(2)-C cells and the otherwise retinoid-resistant IMR-32 line (Rettig et al., 2015). This was accompanied by an increase in the differentiation marker NTRK1, while N-Myc expression was downregulated and tumor growth was markedly reduced *in vivo* (Rettig et al., 2015). Moreover, gene expression analysis confirmed greater neuroblastoma differentiating effects with the combination of multiple HDACi (VPA and SAHA) with either ATRA or isotretinoin versus either compound alone (Hahn et al., 2008). VPA and ATRA combinations induced dramatic morphological neurite extensions and extensive branching as well as greater expression of neurofilament medium (NF-M). In a xenograft model, SAHA-ATRA combination treated tumors had statistically higher differentiation signatures compared to single-agent treated tumors, suggesting that differentiation was in part responsible for the improved survival in the combination treated mice (Hahn et al., 2008).

Treatment with either VPA (Class I and IIa HDACi) or BL1521 (Class I and II HDACi) has also been shown to increase Notch signaling and promote the differentiation of neuroblastoma cells (de Ruijter et al., 2005; Stockhausen et al., 2005). The highly conserved Notch signaling cascade is known to be important in the development of several tissues, including the peripheral and central nervous systems (Artavanis-Tsakonas et al., 1999), and plays a pivotal role in cell fate decisions such as proliferation, stem cell maintenance and differentiation (Bray, 2006).

#### Cell Cycle Arrest

The increase in histone acetylation induced by HDACi is also known to lead to the transcriptional activation of genes associated with either G1 or G2/M cell cycle arrest (Bolden et al., 2006). Accordingly, a number of HDACi have been implicated in promoting cell cycle arrest (Table 1), although it has not been established in all cases whether this occurs in the context of promoting differentiation. For instance, Ouwehand et al. (2005) observed a G1 phase arrest in response to treatment with BL1521, whilst VPA, sodium butyrate, tributyrin have also been shown to induce G2-M arrest (Table 1), all of which have also been shown to also induce differentiation in other studies (Rocchi et al., 1992, 1998, 2005).

Each phase of cell division is promoted by the activity of various cyclin/cyclin-dependent kinase (CDK) complexes, which in turn are tightly regulated by cell cycle inhibitors, such as the Cip/Kip family of cyclin-dependent kinases inhibitors (Abbas and Dutta, 2009). Following the treatment of MYCN single-copy GIMEN and MYCN-amplified SJNB8 cell lines with the HDACi BL1521, CDK4 downregulation, p21 (WAF1/CIP1) upregulation and an increase in the hypo-phosphorylated form retinoblastoma protein was observed (Ouwehand et al., 2005). These findings are consistent with the upregulated gene transcription and protein expression of p21 (WAF1/CIP1) induced by VPA, sodium butyrate, tributyrin (Rocchi et al., 2005) and helminthosporium carbonum (HC)-toxin (Deubzer et al., 2008a,b). On this basis, these results suggest that p21 could be involved in cell growth inhibition and potentially in the induction of differentiation exerted by such HDACi in neuroblastoma cells. This is supported by other reports highlighting the p21-dependent differentiation of leukemic cells by the HDACi VPA (Gurvich et al., 2004). Furthermore, combined treatments of neuroblastoma cells with VPA and the COX-2 inhibitor celecoxib, or either sodium butyrate/vorinostat with the differentiation inducing agent, retinoic acid, also enhanced the induction of p21 (WAF1/CIP1) in neuroblastoma cells when compared to the single agent treatment arms alone (De los Santos et al., 2007; Chen et al., 2011).

The ability of HDACi to promote p21 expression has been observed across multiple cancer types, and also with various different HDACi (Zhao et al., 2006; Vinodhkumar et al., 2008). From a mechanistic viewpoint there may also be a number of different pathways through which HDACi treatment results in this increased expression of p21. This includes the elevated acetylation of p53, which has been observed following treatment with tributyrin (de Conti et al., 2013; Ortega et al., 2016), sodium butyrate (Terui et al., 2003; Ortega et al., 2016), romidepsin (Zhao et al., 2006), VPA (Thakur et al., 2011), vorinistat (Seo et al., 2011; Nebbioso et al., 2017), and entinostat (Miller et al., 2011). As an important tumor suppressor, and key transcriptional activator of p21, the increased acetylation of p53 is quite likely to be involved in the response to HDACi in neuroblastoma. This mechanism may be especially relevant for neuroblastoma, given the low frequency of *TP53* mutations present in these tumors at diagnosis (Schramm et al., 2015). However, the increased prevalency of p53 pathway mutations within relapsed neuroblastoma tumors (Carr-Wilkinson et al., 2010), suggests that HDACi acting via this mechanism may not be as effective as second line treatments.

#### Apoptosis Induction

In addition to inducing cell cycle arrest, HDACi have also been shown to directly activate apoptosis in neuroblastoma cells (Table 1). PARP cleavage, an end stage marker of apoptosis, was found to be induced following BL1521, TSA and romidepsin treatment in a panel of neuroblastoma cell lines (de Ruijter et al., 2004; Panicker et al., 2010; Francisco et al., 2012). One suggested mechanism for the direct activation of apoptosis was the inverse regulation of pro-apoptotic and anti-apoptotic proteins, as combination treatments incorporating HDACi are known to increase the expression of caspases and Bid, and promote the inactivation of the anti-apoptotic proteins XIAP, Bcl-x, RIP, and survivin (Muhlethaler-Mottet et al., 2006). Similarly, the ability of VPA to promote pro-apoptotic neutrophin receptor signaling by upregulating p75NTR and sortilin expression has been identified as an additional mechanism that leads to increased apoptosis of neuroblastoma cells (Dedoni et al., 2020). Pro-nerve growth factor (proNGF) induced activation of p75NTR plays a key role in regulating the survival of neurons and process formation during early development, neuronal death in the developing and aging brain, as well as several neurodegenerative diseases (Dechant and Barde, 2002; Schor, 2005). Prolonged exposure of SH-SY5Y cells to VPA further predisposed the cells to proNGF-induced cell death, triggering apoptosis through JNK-mediated caspase and PARP cleavage (Dedoni et al., 2020). Additionally, studies evaluating the radiosensitization effect of HDACi also observed impairment of DNA repair mechanisms by downregulation of the DNA repair enzyme Ku-86 upon combination with vorinostat (Mueller et al., 2011).

#### Other Non-histone Targets

While originally identified as enzymes that catalyze histone acetylation, a large number of non-histone substrates of HDACs have also been discovered (Glozak et al., 2005), along with observations that specific HDACi can regulate acetylation of these target proteins (Table 1). A key example of this in neuroblastoma comes from the observation that HDACi can alter the activity of MAPK pathways, and thereby modulate cellular processes such as growth, differentiation and apoptosis. In the neuroblastoma cell line SK-N-SH, HDAC4 siRNA inhibition dose-dependently suppressed expression of the MAP2K MKK7, causing a reduction in JNK/c-Jun activity (Wu et al., 2019). Interestingly, MKK7 transcription critically depends on the deacetylation of the transcription factors SP1 and Kruppel-like factor-5 (KLF5) by HDAC4, the inhibition of which is paramount in suppressing the oncogenic JNK/c-Jun cascade involved in glioma cells (Wang et al., 2019). Decreased phosphorylation levels of c-Jun have also been observed in glioblastoma cells following inhibition of HDAC6 (Huang et al., 2020), which is additionally implicated in neuroblastoma tumorigenesis and metastatic dissemination (Subramanian et al., 2011; Zhang et al., 2014). These findings suggest that suppression of MKK7 by the inhibition of class II HDACs, HDAC4 and HDAC6, may represent a promising strategy for preventing JNK/c-Jun cascade-mediated formation of nervous system malignancies.

Another non-histone target, Ku70, was previously identified as a specific HDAC6 substrate (Subramanian et al., 2011). In other studies, HDAC inhibition with TSA was shown to promote the acetylation of Ku70, which drives Bax translocation to the outer mitochondria membrane and triggers the release of cytochrome c and onset of caspase-dependent apoptosis in IMR-32 cells (Subramanian et al., 2005).

*MYCN* is considered the most predominant oncogene in high-risk neuroblastoma and growing evidence suggests that protein-protein interactions between N-Myc and HDACs can cooperate to repress the expression of specific subsets of genes, and thereby enhance cancer cell proliferation and inhibit differentiation. N-Myc has been shown to upregulate HDAC2 gene expression in neuroblastoma cells, and further recruit HDAC2 to the cyclic G2 (CCGN2) promoter, repressing *CCGN2* expression and promoting cell proliferation (Marshall et al., 2010). Additionally, HDAC5 has also been shown to block neuroblastoma cell differentiation and induce proliferation through an interaction with N-Myc (Sun et al., 2014). Taken together, these studies identify HDACs as novel co-factors in N-Myc oncogenesis and provide further support for the potential application of HDACi in the treatment of high risk, N-Myc amplified neuroblastoma. In fact, long term, continuous exposure to panobinostat has already been shown to induce terminal differentiation, a reduction of N-Myc expression and long term survival in tumor bearing TH-MYCN transgenic mice (Waldeck et al., 2016).

In addition to N-Myc, the related c-Myc is also highly upregulated or amplified in ∼10% of high-risk neuroblastoma cases (Zimmerman et al., 2018). Furthermore, the acetylation of c-Myc has been shown to increase upon vorinostat and entinostat treatment of leukemic cells, leading to decreased c-Myc expression and increased TRAIL-mediated apoptosis (Nebbioso et al., 2017). Therefore, while the use of HDACi to treat MYCN-amplified tumors has been previously proposed, their use within this c-Myc amplified/upregulated cohort is also of significant interest.

#### Microenvironmental Effects

The impact of HDACi treatment on the different cell populations that constitute the tumor microenvironment has not been extensively examined in the context of neuroblastoma. However, sodium butyrate, vorinostat, TSA and 4-PB have been strongly implicated in mediating the secretion of the pro-angiogenic factor VEGF (Tang et al., 2004; Muhlethaler-Mottet et al., 2008). Entinostat has also been shown to repress angiogenesis *in vivo* by decreasing tumor vasculature in a neuroblastoma xenograft model (Jaboin et al., 2002). Further to this, the efficacy of antiangiogenic agents is known to be greatly improved when combined with other anticancer drugs (Jain, 2005). Studies evaluating the combination of the angiogenesis inhibitor ABT-510 and the HDACi VPA noted significant reductions in the microvascular density of neuroblastoma xenografts (Yang et al., 2007). In addition, the number of structurally abnormal vessels was reduced, suggesting that the combination of both agents can promote the transient “normalization” of tumor vasculature that has been reported with other antiangiogenic agents and may allow for chemo-sensitization, efficient oxygen exchange and drug delivery (Jain, 2005; Kerbel, 2006).

## Challenges and Resistance Mechanisms

Whilst HDACi safety and efficacy have been evaluated in several pediatric clinical trials (Table 2), little reporting eventuated from these trials, suggesting that a lot more work still needs to be done to develop HDACi for the treatment of neuroblastoma. In particular, pharmacokinetic properties such as poor solubility and relatively short half-life are recurring issues that require further optimization (Konsoula et al., 2009). Strategies aimed at overcoming this issue include the development of HDACi based prodrugs which contain a quiescent compound that is converted to an activated state *in vivo* by enzymatic or chemical reactions (Fan et al., 2020). Considered a promising strategy for clinical optimization, HDACi prodrugs have led to enhanced targeted cancer tissue delivery of HDACi by improving bioavailability, membrane permeability and regulating the half-life and release profile to ensure effective uptake within the nucleus. Despite this, extensive research in prodrug development is required to further understand the physiochemical and biological properties of active agents, as well as the enzymatic and/or chemical mechanisms and observed toxicities that occur during metabolism (Fan et al., 2020).

Our overall understanding of the role of HDACs in cancer and the broad application of HDACi is also continuously evolving. Commonly used broad-spectrum, pan-HDAC inhibitors have been shown to be effective within both *in vitro* and *in vivo* settings, but are not particularly useful for identifying or specifically targeting the particular HDAC isoform that may be responsible for promoting the tumorigenic behavior of neuroblastoma cells. In response to this, considerable efforts are being made to develop isoform-selective deacetylase inhibitors such as tubacin (Lee et al., 2013), which selectively inhibits HDAC6, and PC-34051, a selective HDAC8 inhibitor (Estiu et al., 2010). The development of such isoform-specific HDACi will provide further insights into the specific molecular mechanisms of HDACs in neuroblastoma, and may also provide the specificity required for future precision medicine approaches.

Further to this, rapid transcriptomic or proteomic anlaysis following the over-expression of specific HDACs and transcriptional complexes may provide further insight via systems-level based approaches implemented to predict relevant drug targets and monitor undesired off-target effects (Milazzo et al., 2020). Understanding the structure and functional of these multi-protein HDAC complexes may also allow for the design of novel inhibitors that prevent interactions between protein subunits. A pertinent example of this is the specific targeting of the CoREST complex, which consists of HDAC1/2, the scaffolding protein CoREST, and lysine specific demethylase 1 (LSD1) (Kalin et al., 2018). This was recently achieved through the development of a hybrid agent, Corin, which was derived from the class I HDACi entinostat and an LSD1 inhibitor, and exhibited superior anti-proliferative effects in several melanoma and cutaneous squamous cell carcinoma lines (Kalin et al., 2018). This direction of preferentially targeting epigenetic regulatory complexes through hybrid functionality offers novel and unique therapeutic opportunities over and above mono-functional HDACi.

Resistance to treatment with HDACi is also a growing concern that may impact the therapeutic application of this compound class (Robey et al., 2011). To date, the exact mechanisms underlying resistance to HDACi in neuroblastoma remains largely unknown, although somatic heterozygous mutations in the genes encoding histones H3.1 and H3.3 are observed in pediatric high-grade gliomas (Liu et al., 2014), and truncating mutations of HDAC2 have been attributed to resistance to traditional HDACi in colorectal cancer (Ropero et al., 2006). Findings such as these may prove useful as patient-specific biomarkers that can predict resistance or sensitivity to HDACi (Marks, 2010), although more general mechanisms, such as drug efflux, aberrant cellular antioxidant mechanisms and elevated anti-apoptotic proteins have also been shown to circumvent HDACi-induced cell death across a number of tumor types (Lee et al., 2012). For example, P-glycoprotein (P-gp), which confers resistance by mediating the ATP-dependent efflux of drugs, has been observed at increased levels and implicated in resistance to the HDACi romidepsin in T-cell lymphoma (Piekarz et al., 2004). High levels of the redox-regulating protein thioredoxin are also known to protect transformed cells from oxidative damage by scavenging the reactive oxidative species generated by treatment with HDACi (Marks, 2006; Karlenius and Tonissen, 2010). Fittingly, reduced sensitivity to vorinostat has been correlated with increased expression of thioredoxin in acute myeloid leukemia patients (Garcia-Manero et al., 2008). Additionally, overexpression of the anti-apoptotic survival protein BCL-2 has also been associated with resistance to vorinostat, romidepsin, and panobinostat in patients with cutaneous T-cell lymphoma (Robey et al., 2011), further protecting transformed cells from HDACi-induced cell death. The breadth of these potential resistance mechanisms is possibly a reflection of the plethora of different mechanisms of action for HDACi in neuroblastoma and other tumor types. Therefore, these findings further highlight the need for an increasingly detailed understanding of the specific mechanisms that both emerging, and existing, HDACi utilize to exert their therapeutic benefit in neuroblastoma.

## Concluding Remarks

Whilst HDACi are an emerging class of effective targeted anticancer agents, more work is required to determine how these agents might best be deployed to improve treatment outcomes for neuroblastoma patients. Pre-clinical studies have already provided insight into the histone and non-histone targets of some HDACs in neuroblastoma cells, which together regulate cell proliferation, cell migration and cell death, as well as playing a role in angiogenesis. Clinical analysis has also highlighted the significant correlation between HDAC8 and HDAC10 expression and patient outcome, suggesting their potential utility as predictive biomarkers of therapeutic response to HDACi in neuroblastoma. Compelling evidence also suggests that HDACi may sensitize tumors to current standard-of-care chemotherapy, which could offer urgently needed, improved treatment options for high-risk neuroblastoma patients. In this regard, the timing of HDACi administration relative to treatment with secondary agents should also be considered in the development of effective combination treatments. Given the ability of HDACi to alter the expression of both pro- and anti-apoptotic proteins, priming tumors with HDACi and allowing time for these changes to occur before treatment with chemotherapy may represent an alternative approach that may both maximize efficacy and reduce toxicity (Mohammad et al., 2019). However, to aid the advancement and adoption of HDACi in the clinic, additional work needs to be done to more widely and systematically profile the function of HDACs in neuroblastoma in order to determine the consequences of specific HDAC inhibition in a manner that simultaneously accounts for patient-specific expression profiles, potentially redundant or opposing roles of HDACs and the overlapping specificity of HDACi. Clearly, until both the functional and therapeutic complexities of this enzyme family are fully mapped, their promising clinical utility may not be fully realized.

## Author Contributions

MP performed the literature review and wrote the manuscript. JH performed the literature review and edited the manuscript. YO’D edited the manuscript and prepared the figures. SL conceived the concept and edited the manuscript. DC conceived the concept, edited the manuscript, and supervised MP, JH, and YO’D. All authors contributed to the article and approved the submitted version.

## Conflict of Interest

The authors declare that the research was conducted in the absence of any commercial or financial relationships that could be construed as a potential conflict of interest. The reviewer DD declared a past collaboration with one of the authors DC to the handling editor.
